# Retraction: Evidence That Head and Body Lice on Homeless Persons Have the Same Genotype

**DOI:** 10.1371/journal.pone.0312328

**Published:** 2024-10-14

**Authors:** The PLOS ONE Editors

The *PLOS ONE* Editors retract this article [1, 2] due to concerns about compliance with the PLOS Human Subjects Research policy.

The Methods section in [1] reports that the study involved samples collected in 2010 and that the study protocol was approved by the Institutional Review Board and Ethics Committee of Marseille, under approval number 10.005. A representative of the Aix-Marseille Université Ethics Committee stated that the samples mentioned in the publication are exclusively lice collected in 2010 as part of research authorized by the Comité de Protection des Personnes (CPP); they provided the CPP ethics approval document 2010-A01406-33 which indicates that the CPP approved the study on or after January 24, 2011. PLOS does not accept retrospective ethics approval.

In addition, PLOS identified potential competing interests between members of the Ethics Committee of the Institut Fédératif de Recherche (IFR) 48 that issued document #10.005 and one or more of the article’s authors. Furthermore, the ethics approval numbers cited for this study have been reported in numerous articles (#10.005 in [3–7] and #2010-A01406-33 in [8–27]) which address different research objectives.

The representative of the Aix-Marseille Université Ethics Committee stated that the institute disagrees with the retraction decision, and that their investigation has not determined that the article violates French regulations or international ethics rules. They stated that the study is not considered research involving the human person that would require CPP approval according to French law, because it focused exclusively on the analysis of lice collected previously and there was no specific sampling for this study.

KDMC responded but expressed neither agreement nor disagreement with the editorial decision. AV, RR, PB, and DR either did not respond directly or could not be reached.
